# Forthcoming complications in recovered COVID-19 patients with COPD and asthma; possible therapeutic opportunities

**DOI:** 10.1186/s12964-022-00982-5

**Published:** 2022-11-01

**Authors:** Hadi Rajabi, Deniz Mortazavi, Nur Konyalilar, Gizem Tuse Aksoy, Sinem Erkan, Seval Kubra Korkunc, Ozgecan Kayalar, Hasan Bayram, Reza Rahbarghazi

**Affiliations:** 1 Koç University Research Centre for Translational Medicine (KUTTAM), Koç University School of Medicine, Istanbul, Turkey; 2 Department of Pulmonary Medicine, School of Medicine, Koç University, Istanbul, Turkey; 3 Stem Cell Research Centre, Tabriz University of Medical Sciences, Tabriz, Iran; 4 Department of Applied Cell Sciences, Faculty of Advanced Medical Sciences, Tabriz University of Medical Sciences, Tabriz, Iran

**Keywords:** SARS-CoV-2, COPD, Asthma, Stem cells, Regenerative medicine

## Abstract

Infection with severe acute respiratory syndrome coronavirus 2 (SARS-CoV-2) has been growing swiftly worldwide. Patients with background chronic pulmonary inflammations such as asthma or chronic obstructive pulmonary diseases (COPD) are likely to be infected with this virus. Of note, there is an argument that COVID-19 can remain with serious complications like fibrosis or other pathological changes in the pulmonary tissue of patients with chronic diseases. Along with conventional medications, regenerative medicine, and cell-based therapy could be alternative approaches to compensate for organ loss or restore injured sites using different stem cell types. Owing to unique differentiation capacity and paracrine activity, these cells can accelerate the healing procedure. In this review article, we have tried to scrutinize different reports related to the harmful effects of SARS-CoV-2 on patients with asthma and COPD, as well as the possible therapeutic effects of stem cells in the alleviation of post-COVID-19 complications.

**Video abstract**

**Supplementary Information:**

The online version contains supplementary material available at 10.1186/s12964-022-00982-5.

## Introduction

SARS-CoV-2, a β-type coronavirus, affects nearly all world population after appearing in Wuhan in November 2019 [1, 2]. It was suggested that COVID-19 contributes to extreme clinical symptoms mainly in patients with background metabolic disorders like diabetes and chronic respiratory diseases such as COPD and asthma. The main reason for this claim is the lack of normal function in lung tissue and the existence of chronic inflammation predisposes patients to exhibit subacute and acute forms of COVID-19 [3]. In two studies conducted by different research teams, it has been indicated that SARS-CoV-2 infection is more likely to lead to serious complications in asthmatic conditions and that these patients experience severe forms of COVID-19 with high-rate mortality [4, 5]. Likewise, the entry of pathogenic virus-like human rhinovirus exacerbates asthmatic conditions [6, 7].

Additionally, post-Covid-19 complications are another important issue in COPD and asthmatic patients. It is assumed that individuals affected by asthma and COPD experience serious pathological changes in their respiratory system notably the lungs even after recovery from COVID-19 [8]. In this regard, the restoration of normal lung function in asthmatic and COPD patients, which are affected by COVID-19, is the subject of debate. During the last decades, the application of stem cells and progress in regenerative medicine approaches has provided an alternative therapeutic platform in clinical settings. Considering the differentiation capacity and paracrine activity of stem cells, these cells have been applied for the restoration of injured tissues during several pathological conditions [9]. Here, we summarized reliable data about the effect of SARS-CoV-2 on COPD and asthmatic patients’ health and the possible impact of stem cells in the restoration and alleviation of post-COVID-19-related pathologies.

## SARS-CoV-2 structure and COVID-19 pathogenesis

**Fig. 1:**
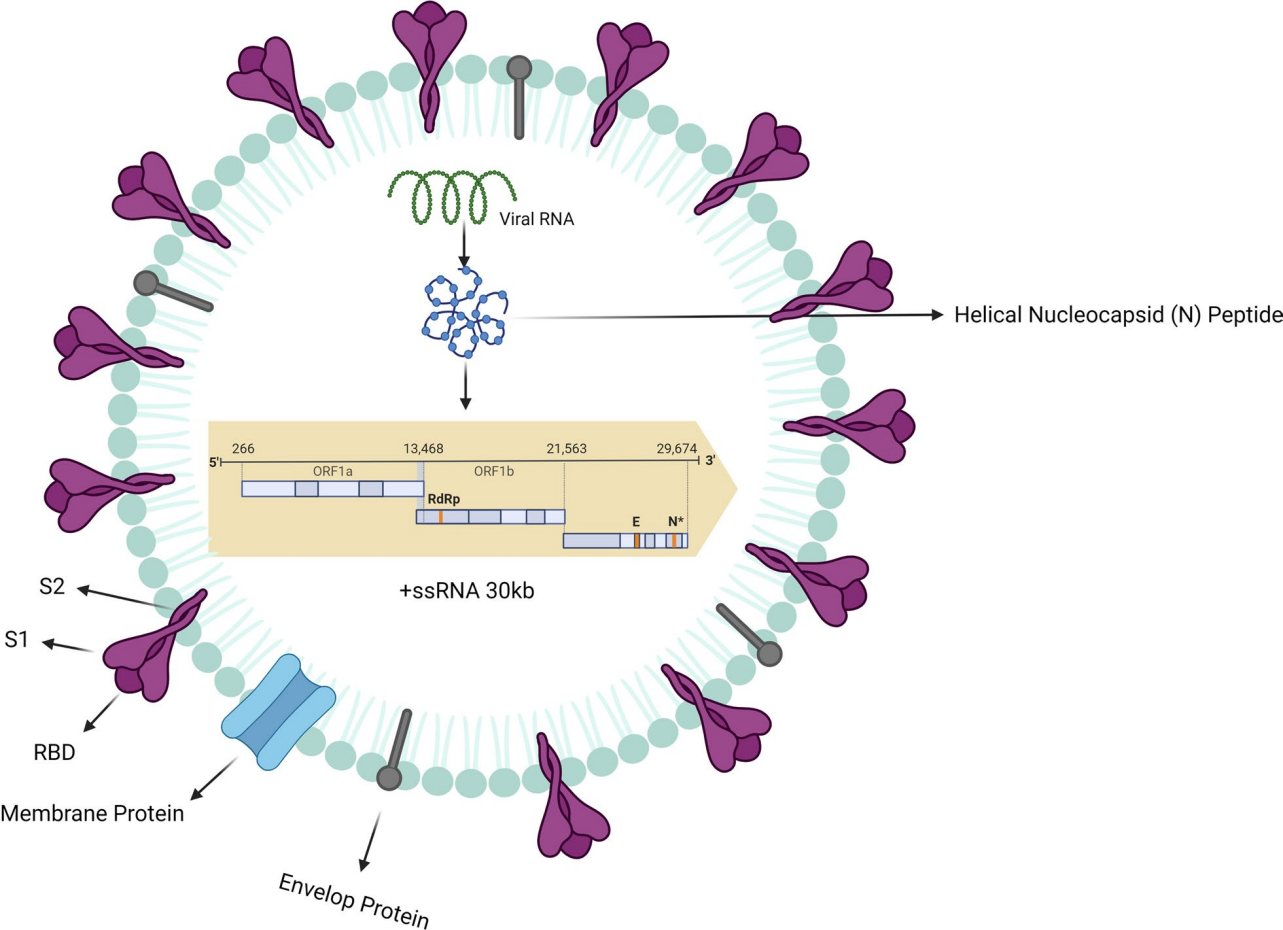
SARS-CoV-2 structure; SARS-COV-2 structural elements include spike protein, envelope, membrane, and internal components such as viral positive single-stranded RNA and nucleocapsid proteins

SARS-CoV-2, a causative agent of COVID-19, was reported first in Wuhan [10]. The human respiratory system is the main target of a virus with a high-morbidity rate compared to the previous forms of other coronaviruses [11]. Molecular investigations have shown that this virus is a new form of β-coronaviruses that was not previously identified in human. The viral nucleocapsid is surrounded by a lipid envelope which is armed with different protein types like a spike (S), envelope (E), and membrane (M) [8]. SARS-CoV-2 with positive-single strand RNA (^+^SSRNA) harbors several open reading frames (ORFs) to encode different viral proteins. ORF1a and 1b are located at the 5’ terminus and involved in the transcription of about 15 different proteins with non-structural (NSPs) and phosphatase (pp1a and b) activity (Fig. 1). NSPs mediate the formation of replication and transcription complex. Besides, the integrity of genome is preserved via NSPs by engaging certain enzymes. Envelop proteins such as M, S, and N are encoded by ORFs at 3’ terminus [12]. Other viral proteins like M and E participate in the shape and formation of the envelope [13].

**Fig. 2:**
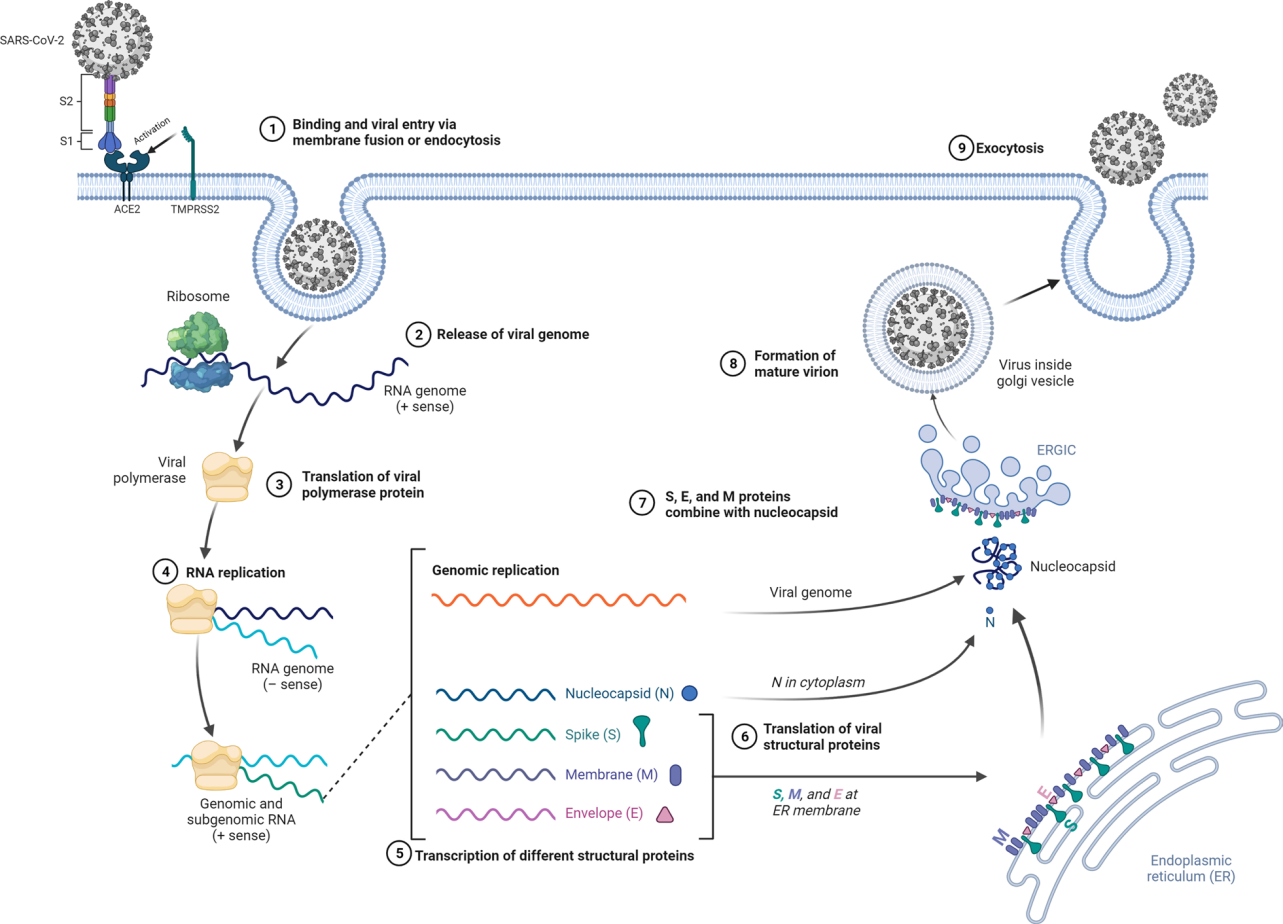
The life cycle of SARS-CoV-2 in host cells; The life cycle of the virus begins with the binding of the S1 protein to the cellular receptor ACE2, followed by proteolytic cleavage that makes the S2 protein facilitate the fusion of the viral and cellular membranes. Within the cell, viral RNA replicates, followed by viral proteins translating from the RNA, followed by the assembly of viral proteins and genome within the endoplasmic reticulum and Golgi. Virions are eventually transported to the cell membrane by vesicles and released by budding from the cytoplasmic membrane

Spike glycoproteins are highly glycosylated and composed of two subunits S1 and S2. These proteins are involved in the attachment of the virus to the host cell angiotensin-converting enzyme-2 (ACE-2) receptor and further entry into the cytosol [14]. S1 subunit exists in trimeric form and initiates virus attachment to ACE-2. Upon physical interaction between spike protein and ACE-2, S1 trimers are decomposed via the activity of cell membrane bonded serine protease (TMPRSS2) and Furin and further secreted into the extracellular matrix (ECM). The process of physical contact is continued via engaging the S2 subunit to progress a fusion step [15–17]. Also, integrins can be acted as alternative receptors for SARS-CoV-2 entry. These proteins can induce a conformational change in viral receptors [16]. Previous studies investigating the entry of SARS-CoV-2 entry into the target cells reported the critical role of the endosomal system in viral replication. The existence of pH-dependent proteases (Cathepsin B and L) inside endosomes can help prime S protein and fusion with the cell membrane [18, 19]. There is direct evidence for the participation of Basigin/CD147 and Neuropilin-1, heparan sulfate, and ADAM17 in SARS-CoV-2 fusion with the host cells [15, 20–22]. Within the cytosol, the released ^+^SSRNA attaches to ribosomes to translate into RNA dependant RNA polymerase for the synthesis of polyproteins, and replications. Later, ORF1a and ORF1b are produced [23]. Following the completion of the translation process, vesicles originated from Golgi apparatus and endoplasmic reticulum Golgi intermediate compartment (ERGIC) transfer the viral components to vesicles to produce virions (Fig. 2) [12]. Once the virus infects the cells the pattern recognition receptors (PRRs) detect the viral genome.

ACE-2 receptor is mainly localized at the apical surface of epithelial cells in pulmonary and gastrointestinal tracts, kidneys, and cardiac tissue [24]. It is believed that both ACE-2 receptor and TMPRSS2 can be detected almost in all cell types within the pulmonary tissue even in cells isolated from subsegmental bronchial branches [25]. The cellular distribution of TMPRSS2 is relatively equal in both lungs and subsegmental bronchial branches while the content of ACE-2 receptor is high in secretory cells [25]. The expression of the ACE-2 receptor and TMPRSS2 is high in type II alveolar cells (pneumocytes) compared to type I alveolar cells. In line with these statements, type II alveolar cells along with secretory cells are the main cellular targets for SARS-CoV-2 [13].

Inflammation is an investable procedure and is actively implicated during the infection of tissues with SARS-CoV-2. The close interaction between macrophages and SARS-CoV-2 can contribute to macrophage frustration. Then, the release of several cytokines by activated macrophages primes T lymphocytes such as T helper type 17 (Th17) cells. Recent works have revealed that a large number of factors like tumor necrosis factor-β (TNF-β), interleukins (IL)-1, -6, -8, and -21, monocyte chemoattractant protein-1 (MCP-1), CXCL10, and CCL2 are specifically present in the secretome released by activated macrophages [26]. Remarkably, these cytokines can lead to vasodilation and vascular permeability after leaking into the circulation. Importantly, weakening vascular integrity promotes plasma leakage into interstitial parenchyma and alveolar space, leading to an impaired gas exchange [27–29]. The activation of Toll-like receptors (TLR-3, and -7) can result in the detection and entry of SARS-CoV-2 into the target cells. Activated TLR-3 and -7 stimulate the phosphorylation of downstream effectors which activate NF-κB. The current understanding of NF-κB activity implies that this mechanism is required for the production of nuclear IFN and other inflammatory factors. An increase in type I IFN appears to eliminate internalized viruses. While the suppression of IFNs can trigger overproduction of cytokines and causes cytokine storm syndrome [30]. It is important to keep in mind that during this phenomenon an array of several cytokines like IL-1, -6, -17, -21, and -22, and TNF-α is released. In the latter infection stages, the elevation of these mediators recruits further neutrophils and macrophages via the modulation of CD4+ Th1cells activity. The continuity of these responses makes breathing difficult with remarkable hypoxemia and coughing [30, 31]. Local production of cytokines and continuous recruitment of immune cells into the infected sites lead to acute respiratory distress syndrome (ARDS) and organ failure.

## Pathophysiology of COPD and asthma

### Asthma

**Fig. 3:**
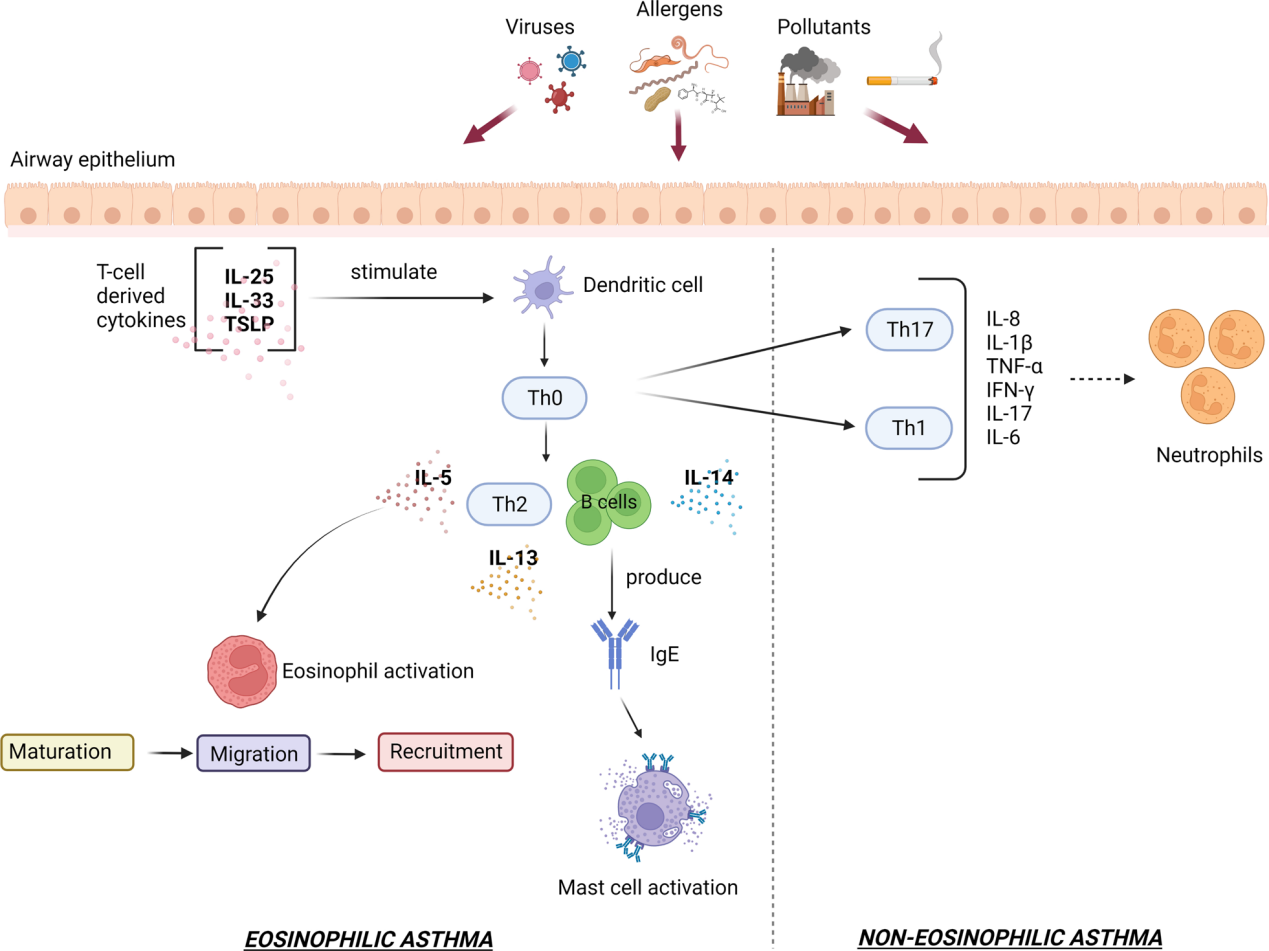
Schematic of eosinophilic and neutrophilic asthma immunopathology. In eosinophilic asthma, type 2 T helper cells secrete T cell-derived cytokines (TSLP, IL-25, and IL-33), which results in the recruiting of eosinophils. TSLP stimulates local dendritic cells, Activation of dendritic cells leads to the differentiation of lymphoblast toward type 2 T helper cells via presenting antigens to type 1 T helper cells. Type 2 T helper and B cells activation contribute to the production of IL-4, -5, and-13. B cells produce a large content of IgE, finally leading to mast cell activation. On the other hand, in non-eosinophilic asthma, the release of IL‐1β, -6, -8, -17, IFN‐γ, and TNF‐α by type 1 and 17 T helper cells cause neutrophils recruited into the pulmonary niche. *Th* T helper cells (0, 1, 2, and 17), *TSLP* Thymic stromal lymphopoietin, *IL* interleukin

**Fig. 4:**
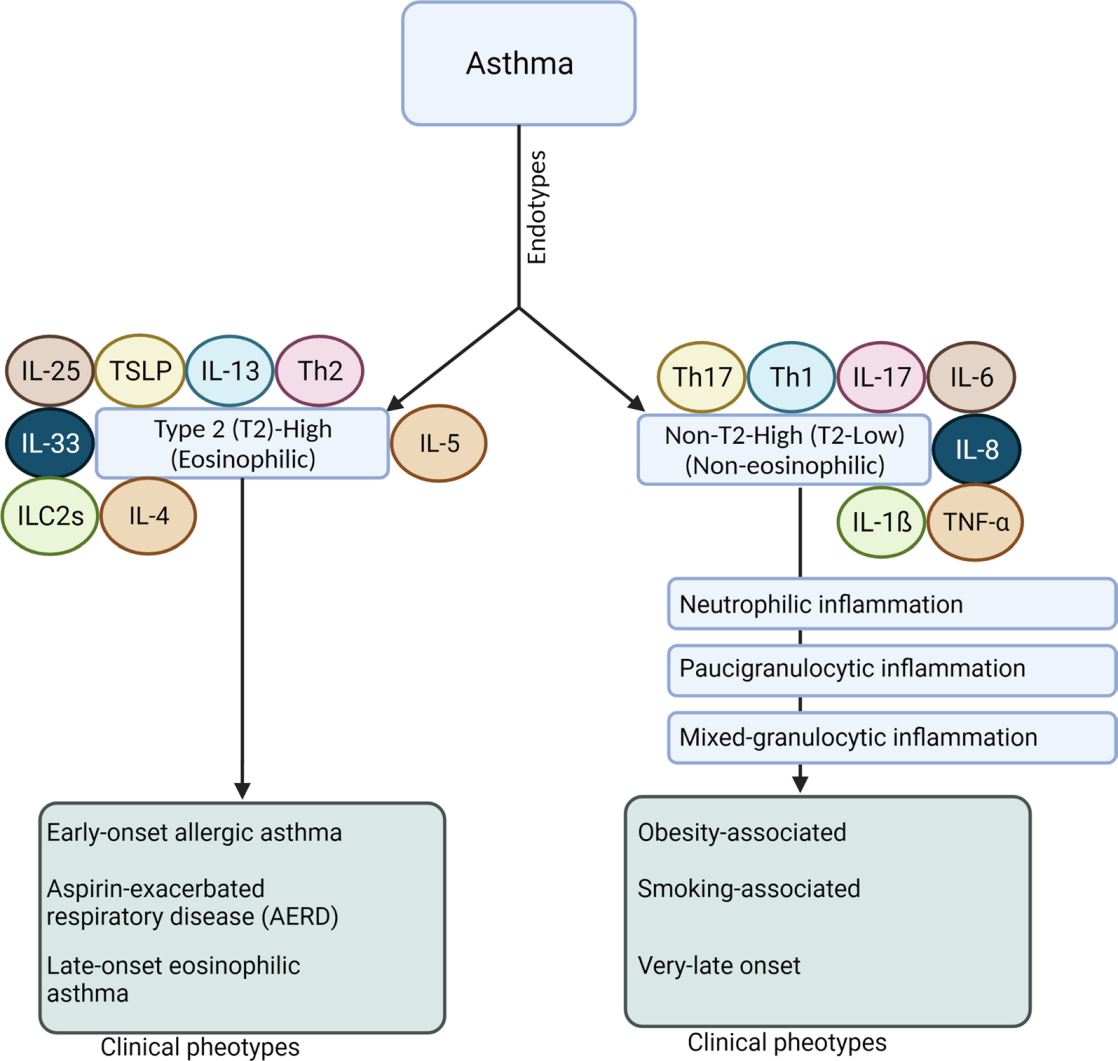
Flowchart for the endotypes and clinical phenotypes of asthma. *IL* interleukin, *ILC* interleukin-like cells, *Th* T helper cells, TNF-α tumor necrosis factor-alpha

Asthma is a chronic inflammatory condition affecting both conducting and respiratory zones. In asthmatic patients, airway hyperresponsiveness and massive remodeling of lung parenchyma are the main pathological findings [32]. It has been shown that both genetic and environmental factors such as viruses, allergens, or occupational irritants have a role in this disease’s evolution [33, 34]. In individuals being exposed to these factors, airway inflammation is induced which is underlying asthma pathogenesis. Under normal conditions, the airway inflammatory response is tightly regulated by the balance between effector and regulatory immune cells [35]. With the onset of asthma, several immune cell types such as mast cells, eosinophils, Th2 cells, basophils, and platelets are abnormally recruited into target sites within the pulmonary niche (Fig. 3) [36–39]. The production and release of several chemokines and cytokines such as histamine, leukotrienes, prostanoids, kinins, and platelet-activating factors from immune cells can deteriorate the physiological behavior of epithelial cells, smooth muscle cells, vascular cells, and local dendritic cells. Along with these changes, the occurrence of massive pathological remodeling activates the dynamic growth of fibroblasts [35, 40–42]. In many cases, histological examination shows a dominant eosinophilic, neutrophilic, and/or mixed eosinophilic-neutrophilic inflammatory response. Based on the eosinophilic response, data suggest two asthmatic conditions including eosinophilic [high T2/T1 cell ratio and eosinophilic reaction] and non-eosinophilic [low T2 helper cells with a low number of eosinophils] types (Fig. 4) [43]. In eosinophilic asthma, Th2 cells secret certain cytokines such as thymic stromal lymphopoietin (TSLP), IL-25, and IL-33 to recruit eosinophils [35, 43]. This form of asthma is clinically characterized by an early-stage allergic (atopic) reaction, leading to the dysregulation of the airway epithelial cell barrier. T cell-derived cytokines mainly TSLP can also stimulate local dendritic cells which in turn intensifies the eosinophilic reaction at the target sites [44, 45]. Further activation of dendritic cells can increase differentiation of lymphoblast toward Th2 cells via presenting antigens to Th1 cells [46]. In this scenario, Th2 and B cell activation can contribute to the production of IL-4, -5, and -13 [47, 48]. Based on these events, B cells can mature into plasma cells and produce a large content of IgE, leading to mast cell activation [47]. In contrast to eosinophilic (atopic) asthma, in non-eosinophilic form types, Th1 and Th17 cells along with neutrophils play a critical role in the progression of pathological changes. Data suggested the presence of different inflammatory factors such as IL‐1β, -6, ‐8, 17A/F, IFN‐γ, and TNF‐α [49]. The most prominent cellular event is likely a neutrophilic reaction and is seen in smokers and individuals with diabetic changes [43]. Activation of TLRs and release of IL-1β, -8, and TNF-α by Th1 and Th17 cells cause neutrophils recruited into the pulmonary niche [50]. The second type of non-eosinophilic asthma is known as paucigranulocytic asthma which is determined by the lack of changes in sputum or blood levels of eosinophils or neutrophils [51]. Data suggested the lack of prominent inflammation thus it is thought that this type of asthma closely correlates with dysfunction or abnormal morphology of smooth muscle cells, and nervous and vascular tissues [52, 53]. In case, inflammatory responses occur both neutrophilic and eosinophilic reactions (mixed granulocytic asthma) can be detected [54].

### COPD

**Fig. 5:**
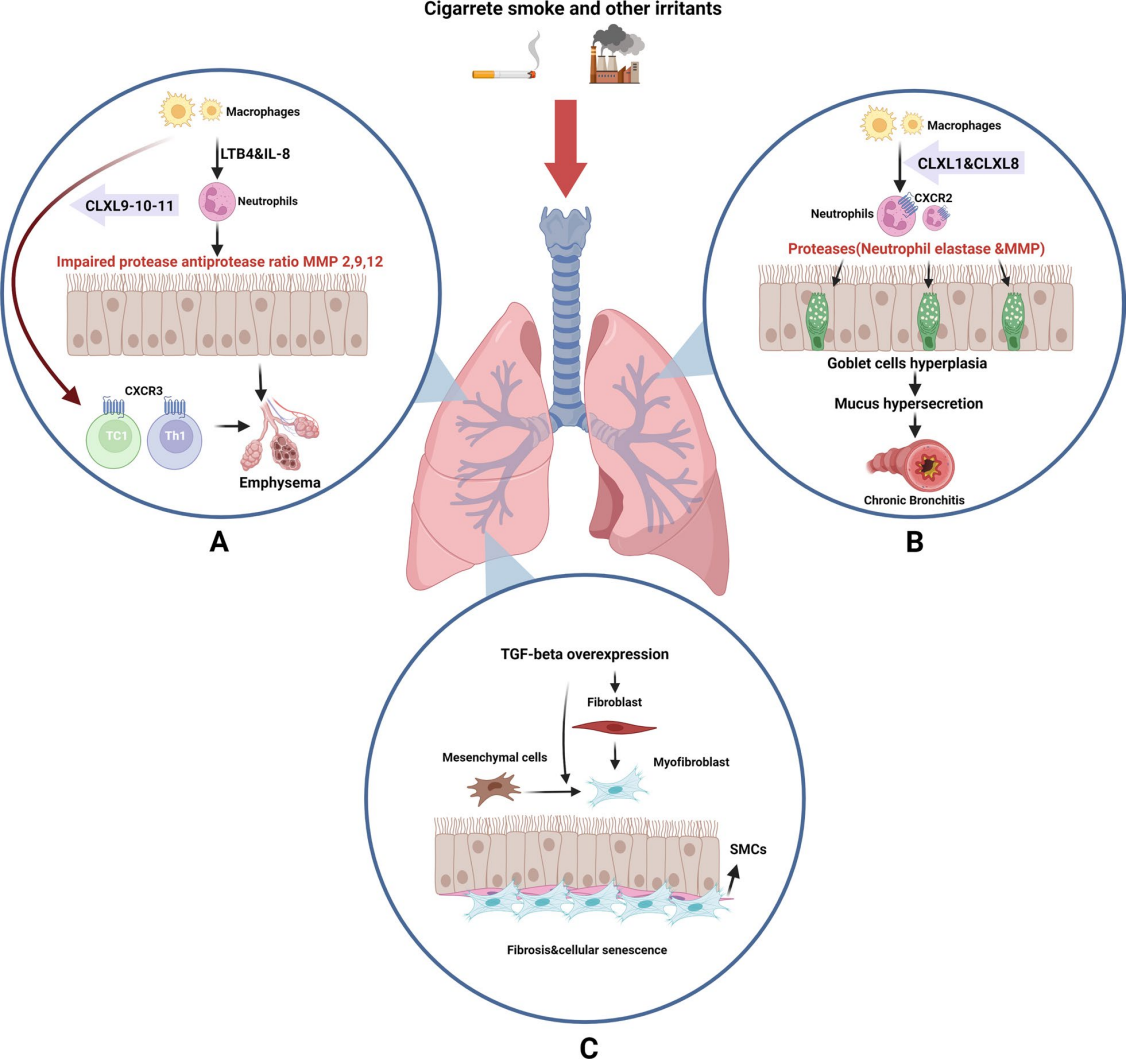
The COPD etiology is depicted in the diagram. In addition to activating epithelial cells, cigarette smoke and other environmental stimuli can also recruit neutrophils and macrophages from the bloodstream, which release a variety of chemotactic signals that recruit inflammatory cells to the lung. For instance, the CXC-chemokine receptor (CXCR) 2 is used by CXCL1 and CXCL8 to recruit neutrophils and monocytes. CD+ 8T cells can be recruited by CXCL 9, 10, and 11. The MMPs (2, 9, and 12), elastase, and Cathepsin K, L, and S that are involved in lung fibrosis and emphysema are also secreted by activated lung epithelial cells

Like asthma, COPD is a chronic inflammatory disease characterized by bronchitis, bronchi thickening, and emphysema [55, 56]. In response to inhaled particles, the injury and activation of epithelial cells lead to the infiltration of neutrophils and monocytes from blood into the pulmonary system. In collaboration with alveolar macrophages (dust cells), immune cells produce cytokines, chemokines, devastating enzymes, and free radicals (ROS) resulting in alveolar tissue remodeling (Fig. 5). Subsequent activation and recruitment of dendritic cells into the bronchi elevate in situ number of Th 1 and Th 17 cells. The addition of T cytotoxic lymphocytes (CD8^+^ cells) leads to the massive destruction of alveolar cells. It is proposed that the presence of autoantibodies in bronchoalveolar lavage of patients with COPD shows B cell activation [57, 58]. As a consequence, these changes activate compensatory responses which are linked to mucus hypersecretion, lung emphysema, pulmonary hypertension, and cor pulmonale [59]. An excessive mucin production promotes cell metaplasia in airway conduits. To be specific, the number of goblet cells increases in response to chronic conditions coincides with the hyperplasia of submucosal glands [60]. Phenotypic and morphological changes mediated by metaplasia can alter the length of cilia via a proteolytic process called ciliophagy [61]. As the number and length of cilia decreased, the mucus movement is interrupted through the pulmonary tract. Besides, the progress of metaplasia would exacerbate mucus entrapment [62]. This phenomenon can result in obstruction of airway conduits with a size of less than 2 mm. The loss of lung elasticity and emphysematous changes can accelerate the obstruction of airway conduits, leading to lung hyperinflation. The resulting high CO_2_ and low O_2_ concentrations in the blood of COPD patients contribute to hypercapnia and hypoxemia, respectively.

## Post-COVID complications in patients with background COPD and asthma

Whether background chronic pulmonary diseases can increase the possibility of COVID-19 in these patients is at the center of attention and needs further studies [63]. It has been shown that the occurrence of chronic pathological conditions such as COPD, asthma, smoking etc. can increase the severity and morbidity of COVID-19. These conditions enhance the susceptibility of respiratory conduit to viral infections such as SARS-COV-2. Even after recovery from COVID-19 infection specific pathologies such as fibrosis and massive tissue remodeling are mighty [64, 65]. In support of this notion, the regulation of receptors like ACE2- and CD147-related genes is induced in bronchial cells under chronic pathological conditions [63]. As above-mentioned, over-production of mucus in COPD patients can lead to respiratory distress and activation of inflammatory cascade which in turn contributes to the cytokine storm [66]. Besides, the normal activity of the mucociliary system is blunted in individuals with COPD, making these people more vulnerable to COVD-19 infection [67]. Despite this claim, some contradictory evidence has also been reported. Interestingly, some studies reported a low rate of COPD in COVID-19 patients [63]. One reason would be that the prolonged administration of corticosteroids and bronchodilators in COVID-19 patients can reduce the risk of COPD [63]. On the other hand, the hyperactivity of goblet cells and enhanced mucus secretion can be a physical barrier to reducing the accession of viral particles to the bronchial layer and alveolar cells [63]. Despite these facts, there is not enough proof for this hypothesis [68]. In addition to the remaining side effects of SARS-COV-2 infection in patients with COPD, also the mortality rate is significantly higher in these patients. The precise molecular and cellular mechanism supporting high-rate mortality in COPD patients after infection with SARS-COV-2 needs to be elucidated. Data suggest that COPD and such chronic conditions can increase the inflammatory burden, leading to significant mortality rates after infection with SARS-CoV-2 [69, 70]. The promotion of significant pathological changes under chronic conditions is linked to disruption of the mucociliary system and weakened function of alveolar macrophages [71, 72]. Even though, the number of alveolar macrophages increases in COPD patients. Lower contents of IRF-7, INF-α, and -β in COPD patients and smokers are the main cause of susceptibility to COVID-19 [73]. These factors are known to reduce SARS-CoV-2 replication inside the cytosol. In COPD patients, the activation of the ADAM17/EGFR axis can be initiated via the TLR signaling pathway. Both pro- and anti-inflammatory responses can be triggered via the activation of the ADAM17/EGFR axis. The production of CXCL8 is associated with neutrophil recruitment into the pulmonary niche. On the other hand, prolonged activation of the ADAM17/EGFR axis can cause anti-inflammation via the shedding of TNF-R2 [74]. Along with this claim, there is an abnormality in the function of adaptive systems such as B and T lymphocytes. Lower contents of polymeric immunoglobulin receptors also raise the possibility of IgA suppression and lack of appropriate viral clearance. The continuity of chronic immune responses can contribute to exhaust CD8^+^ lymphocytes and suppressed T regulatory cell function [73].

Quantitative transcriptomic analyses have revealed an apparent upregulation of ACE-2, TMPRSS2, and Furin in the pulmonary tissue of COPD and smokers. To this end, it is postulated that there is a close association between COVID-19 mortality and chronic respiratory diseases [75–77]. With regards to significant changes in the expression of key factors required for SARS-CoV-2 replication and deficient immune response, one can hypothesize that the occurrence of COVID-19 disease can cause more severe clinical manifestations and post-complications in COPD and smokers [78]. Results showed moderate to high persistent lung abnormalities in one out of third patients after infection with SARS and/or MERS [78]. Whether and how COVID-19 infection can cause similar outcomes in the target population needs further investigation. Evidence point to the high possibility of permanent pathological remodeling in the lung parenchyma, and chronic inflammation in COPD patients after infection with SARS-CoV-2, leading to chest pain, fatigue, and breathlessness [79]. Results have shown abnormal proliferation of local progenitors and goblet cells with concomitant ciliary dysfunction [80]. The lack of appropriate epithelialization rate and disruption of the mucociliary system cause microbial overload and vulnerability to viral infection. Endothelial layer damage and aberrant vascular remodeling reduce the number of recruited circulating stem cells into the pulmonary niche, resulting in local stem cell exhaustion, cellular senescence, and reduced regeneration capacity [81–83].

Based on previous data, asthmatic patients are also vulnerable to SARS-CoV-2 infection [84]. Patients with asthma experience deficiency in the innate immune system and anti-viral defense mechanism. These features intensifies cytokine storm in conditions associated with COVID-19 infection [85]. It has been confirmed that antiviral IFN response is significantly impaired in asthmatic conditions, which allows SARS-CoV-2 to easily enter target cells [86]. Like COPD, the onset of asthmatic condition up-regulates the expression of TMPRSS2 in human bronchial epithelial cells and increases SARS-CoV-2 infectivity due to acceleration of the spike protein cleavage [65]. Given the highly intricate nature of immune cell response within asthmatic parenchyma, the possible inhibitory effect of eosinophils and Th2 cell activity should be addressed regarding SARS-CoV-2 infection [87]. Clinical observations have confirmed the more severe form of COVID-19 disease in asthma patients [88]. These findings support the fact that stimulation of effectors associated with virus replication can overcome anti-inflammatory responses driven by recruited cells. In addition to immune cell exhaustion, findings direct to infection of these cells with viral particles. For example, CD147, also known as the Basigin protein, acts as a potent receptor for SARS-CoV-2 internalization in T lymphocytes and some epithelial cell types [65]. Other extracellular proteins such as Cyclophilins A and B, and CD44 can activate CD147 [89, 90]. It implies that the levels of CD147 and Cyclophilin B are elevated during asthmatic conditions [65]. Interestingly, it has been shown that Cyclophilin B is co-expressed with ACE-2 receptors under asthmatic conditions [65]. Molecular investigations have revealed an inverse correlation between ACE-2 receptor expression in the apical surface of epithelial cells and IL-4, -5, and -13 levels. In contrast, the elevation of these cytokines induces TMPRSS2 [91]. Compared to eosinophilic asthma, the levels of TMPRSS2, ACE-2 receptor, and furin are significantly higher in neutrophilic asthma [92]. Like COPD, it seems that background asthma makes patients vulnerable to post-COVID-19 symptoms such as shortness of breath [93].

## Cell therapy in COPD and asthma

There are various approaches for the application of stem cells in varied pathological conditions. These cells can be directly used as naive cells or differentiated by-products after in vitro pre-treatments (Table 1). Differential self-renewality and differentiation capacity make stem cells unique cellular elements for the acceleration healing procedure (Table 2). From the aspect of differentiation capacity, stem cells include embryonic stem cells (ESCs), induced pluripotent stem cells (iPSCs), and mature types like mesenchymal stem cells (MSCs) [94–96]. ESCs can be isolated from the inner cell mass of blastocysts. These cells exhibit high-rate proliferation and bulk capacity to commit into three germ layers [97]. Currently, ethical criticism and the possibility of alloreactive immune cell activity have limited the extensive application of these cells in human subjects. These cells can be used as an allogenic cell source after differentiation into adult stem cell types [98, 99]. iPSCs, with stemness features similar to ESCs, are generated from somatic cells after genetic manipulation without human embryo samples [100]. These cells can help the resident tissue cells to heal the injured areas [100, 101]. In addition to the application of iPSCs as cell-based transplants, their secretory elements such as exosomes (Exo; nanovesicles with an average size of 40–150 nm) have been used for the alleviation and regeneration of injured myocardium because of angiogenic, anti-immunogenic, anti-fibrotic, and anti-apoptotic potential [102, 103]. MSCs are widely used, because of their easy expansion and non-invasive sampling [104]. Besides, these cells can be isolated from varied tissue types such as bone marrow, adipose tissue, umbilical cord, and amniotic fluid [105, 106]. Minimum levels of major histocompatibility complex (MHC-I and II), and other co-stimulatory factors with prominent immune regulatory features make MSCs the most accessible cell source for transplantation [107, 108]. Owing to differentiation capacity, and genomic stability, MSCs have been used for several pathological diseases [109, 110].

**Table 1:** Clinical approaches in using stem cells in different disorders

Disease	Source	Model	Type of administration	Results	References
Multiple sclerosis	ESCs	In vivo	Cell injection	Promote remyelination	[134]
Ischemic reperfusion injury	IPSCs	In vivo	Intramyocardial (48 h after reperfusion)	Promote angiogenesis Ameliorate apoptosis and hypertrophy. No effect of infarct size	[135]
Acute myocardial infarction	IPSCs	In vitro	–	Restore post-ischemic contractile performance, ventricular wall thickness, and electrical stability	[136]
Myocardial infarction	IPSCs-derived cardiomyocytes	In vivo	Intramyocardial, cell injection	Improve cardiac function	[137]
Diabetes mellitus	Mouse skin-derived IPSCs	In vivo	Cell injection	IPSCs differentiate into β-like cells, and these could secrete insulin in response to glucose and correct a hyperglycaemic phenotype	[138]
Parkinson’s disease	IPSCs	In vivo	Cell transplantation	After the transplantation, the cells differentiate into glia and including glutamatergic, GABAergic, and catecholaminergic subtypes. IPSCs were induced to differentiate into dopamine neurons of midbrain character and could improve behavior	[139]
Stroke	BMSCs	In vivo	Intravenous	Enhance rotarod and adhesive removal	[140]
Crohn’s disease	Adipose-derived-MSCs	In vitro	–	Impair T helper type-1 cell activation and expansion of CD4+ CD25+ forkhead box (FOX) P3+ T-regulatory cells that suppress T-helper type 1 effector responses	[141]
Asthma	Human MSCs	In vitro	–	MSCs can suppress proliferation and effector function of CD4+ Th2 cells, Immunoglobulin-E (IgE) production in plasma cells, and IgE-dependent activation of mast cells	[142]
Systemic lupus erythematosus	Mouse MSCs	In vivo	Tail vein injection	It can suppress Th17. Also, MSCs increase CD4, CD25, FoxP3, and Tregs	[143]
Lung injury	Mouse MSCs	In vivo	Jugular Vein injection	MSCs support TNF-α oscillation and they alienate the IL-1a function	[144]
Parkinson diseases	Rat MSCs	In vivo	Intranasal injection	In Parkinson's disease, MSCs reduce inflammatory cytokine secretion	[145]
Liver fibrosis	Mouse MSCs	In vivo	Tail vein injection	The secretion of MMP-9 and MMP-14 went up. Also, it reduced TGF-β1	[146]
Myocardial infarction	Human MSCs	In vivo	Transendocardial injection to pig	They triggered endogenous cardiac stem cells	[147]
Skin wound	Human MSCs	In vivo	Tail vein injection to mouse	MSCs compress Th17 cells and enhance the expression of IL-10	[148]
Corneal abrasion	Mouse MSCs	In vivo	Intravenous and intraperitoneal	MSCs support the production of TSG-6 that is a type of anti-inflammatory protein	[149]
Rheumatoid arthritis	Human MSCs	In vivo	Intraperitoneal injection to mouse	MSCs can go down the cell effusion of Th1 and Th17,	[141]
Graft-versus-host disease	Mouse MSCs	In vivo	Intravenous	MSCs reduce TNF-α, IFN-γ, and IL-12 which are a type of inflammatory cytokines	[150]
Acute lung injury	Mouse MSCs	In vivo	Intravenous	MSCs hinder Th2-moderated allergic airway inflammation	[151]

*ESCs* embryonic stem cells, *IPSCs* induced pluripotent stem cells, *BM-MSCs* bone marrow-derived mesenchymal stem cells, *MSCs* mesenchymal stem cells, *Th2 cells* T helper 2 cells

**Table 2:** Different mechanisms are involved in stem cell regeneration ability

In vivo/in vitro* or* disease models	Type of stem cells	Involving factors	Outcome	Ref
Rat’s acute lung injury	BM-MSCs	Downregulation of inflammatory factors: VEGF, NF-ƘB, and IL-17A	Alleviating lung injury	[152]
			Repairing of alveolar type II epithelial cells	
Lung progenitor organoid cultures	BM-MSCs	Increasing the number of Epcam + Sca-1 + distal lung epithelial cells	Increased alveolar differentiation to tissue repair	[153]
COPD patients' tissue-derived organoids/A549 cell line	Lung derived MSCs	Downregulation of TGF-β and WNT-5A	Induce generation of alveolar organoids	[154]
			Attenuates alveolar epithelial injury	
			Trigger Epithelial repair	
Rat myocardial infarction model	BM-MSCs	Increasing the expression of TGF-β, FGF-2, angiopoietin-2, VEGF-1	Increase in angiogenesis rate	[155]
			Induces myocardial regeneration and cardiac function	
The 4-week-old female New Zealand white rabbits injured endometrium model	BM-MSCs	Increasing CK-19 expressions	Regulate repair of injured endometrium	[156]
		Upregulation of TGF-β1, TGF-β1R, and Smad2 mRNA	Enlarging the number of glands of the endometrial damaged uterus	
		Downregulation of the expression of TGF-β1 and Smad2 mRNA	Reducing e fibrosis area of the endometrial damaged uterus	
Culture of human iPSC-MSCs Bowel disease models in mice	iPSC-MSCs	Multiplying the frequency of CD44+ cells and CBC stem cells (Lgr5+ cells) in colonoids	Promote crypt epithelial cell proliferation via TSG-6	[157]
		The expression and secretion of TSG-6 before the coculture with colonoids		
Mice DSS (dextran sulfate sodium)-induced colitis model	ESC-MSCs	Decrease of CXCL1, CXCL2, IL-6, and MCP-1 significantly	Ameliorate colon epithelial proliferation and integrity	[158]
		Reducing epithelium loss and inflammatory cell infiltration	Encourage the colon epithelial integrity and regeneration	
		Increase of IGF1R, p-IGF1R, AKT, and p-AKT in the treatment group		
Sprague–Dawley rats cutaneous wound healing models	HucMSCs	Increasing B-catenin and its downstream genes (cyclin-D1, cyclin-D3, and N-cadherin)	Promote wound closure of reversible scratches with concomitant treatment	[159]
		The induction of b-catenin downstream genes (cyclin-D1, cyclin-D3, and N-cadherin)	Upgrade the phosphorylation of GSK3b and inhibited the activity of GSK3b	

In the context of pulmonary diseases, stem cell therapy has led to the alleviation of several pathological conditions such as COPD and asthma in animal models [111]. Researchers have applied MSCs in several animal models of asthma types such as non-allergic, allergic, and cough-variant forms [112]. It was suggested that MSCs isolated from bone marrow, adipose tissue, umbilical cord blood, and placenta present a potential to diminish airway hyperresponsiveness and immune cell infiltration in animals with the acute model of asthma [113, 114]. It seems that the source of MSCs is important in achieving regenerative outcomes. For example, Abreu et al. claimed that bone marrow MSCs (BM-MSCs) have the superiority to reduce asthma pathologies such as fibrosis, eosinophilic reaction, and respiratory injury when compared to MSCs isolated from lung adipose tissue[115]. Systemic transplantation of BM-MSCs via tail vein reduced bronchiolar hypersensitivity, neutrophilic response, and Th17 cells cytokines in animal models [116].

Like asthma, numerous studies have examined the therapeutic effects of MSCs in COPD. A meta-analysis study revealed that both intravascular and intratracheal administration of MSCs can be in favor of blunting acute pulmonary inflammation and reduction of apoptotic changes in animals [117]. Intravenous injection of MSCs can promote abnormal vascularization induced by emphysematous changes in animal models [118]. In a similar work, MSCs ceased the production of pro-inflammatory cytokines and increased epithelial growth factor, accelerating the regeneration procedure with two side activities [119]. In a clinical trial study, intravenous injection of allogenic MSCs in COPD patients diminished acute phase protein levels such as C-reactive protein up to 1 month after transplantation without significant differences in pulmonary function tests [107, 120, 121]. Despite the beneficial properties of MSCs in pulmonary diseases, there is still uncertainty about their application as the therapeutic choice in the clinical setting.

## Stem cells in COPD and asthmatic patients with COVID-19 post-complication

As above-mentioned, COPD and asthma patients are vulnerable to infection with SARS-CoV-2 and its post-complication indicated persistent lung injury. Despite these descriptions, stem cell therapy might be a choice for the alleviation and promotion of the healing process in these patients. Of note, in an experiment iPSC-derived type 2 alveolar cells were administrated via the systemic route in a mouse model of hyperoxia, a condition that can be seen in severe COVID patients. These cells successfully diminished hyperoxia-induced alveolar injury. The main reason for the application of iPSC after differentiation to type 2 alveolar cells is that this strategy would decrease the possibility of teratoma development and immune cell infiltration [122]. In another study, direct administration of iPSCs and a step-wise differentiation procedure restored the function of the mucociliary system via differentiation into multiciliated cells [123]. Takuji and co-workers produced functional alveolar macrophages from iPSCs to re-establish immune function within the lung parenchyma [124]. In a similar work, Happle et al. used iPSC-derived macrophages in a mouse model of pulmonary alveolar proteinosis. Results indicated the reduction of alveolar proteinosis, surfactant protein D ratio, and broncho-alveolar turbidity [125]. However, there are no specific clinical trials associated with the direct application of iPSC-derived macrophages and/or goblet cells in COVID-19 cases. Based on data from animal models and in vitro studies, it is assumed that the administration of differentiated cell types via different injection routes may bring regenerative outcomes. Due to the persistent and massive fibrosis in pulmonary parenchyma of severe COVID-19 survivors [126–128], the application of stem cells and/or mature cell types is directed to inhibition of fibrosis, in case the cell therapy is the only choice. In support of this notion, iPSC-derived type 2 alveolar cells alleviated bleomycin-induced fibrosis in a rat model via the inhibition of TGF-β and α-smooth muscle actin [129]. Likewise, these cells are potent enough to suppress epithelial-mesenchymal transition induced by TGF-β [130].

More recently, MSCs and Exo have been applied to patients with SARS-CoV-2, leading to hopeful results (Table 3). Intravenously injected MSCs decreased lung inflammation, improved clinical symptoms, and returned oxygen saturation to normal levels [131]. In phase 1 clinical trial study, Wharton's jelly MSCs exhibited prominent anti-inflammatory features with the elevation of IL-10 and stromal cell-derived factor-1. Besides, the levels of pro-inflammatory cytokines were reached near-to-normal levels. Despite the existence of promising outcomes, headache in one of the patients has been reported which alleviated without any pharmaceutical intervention [132, 133].

**Table 3:** Some list of clinical trials in using stem cells in Covid-19 patients recorded up to April 2022 (available at https://clinicaltrials.gov/us)

Status	Study	Stem cells type	Condition	Phase
Recruiting	Mesenchymal stem cell infusion for COVID-19 infection	BM-MSCs	Covid-19	Not applicable
Recruiting	Safety and efficacy study of allogeneic human dental pulp mesenchymal stem cells to treat severe COVID-19 patients	Allogeneic human dental pulp stem cells	Covid-19	Not applicable
Completed	Study evaluating the safety and efficacy of autologous non-hematopoietic peripheral blood stem cells in COVID-19	Autologous non-hematopoietic peripheral blood stem cells	Covid-19	Not applicable
Completed	Mesenchymal stem cell secretome in severe cases of COVID-19	MSCs secretome	Covid-19	Not applicable
Completed	Menstrual blood stem cells in severe Covid-19	Allogeneic human menstrual blood stem cells secretome	Covid-19 cytokine storm	Not applicable

## Conclusion

The SARS-CoV-2 virus can cause extreme complications either during the infection or after remediation in COPD and asthmatic patients. It is logical to hypothesize that post-COVID complications can be more probable and last for a long time in these groups of patients. However, stem cell-based regenerative medicine with the capacity to recreate cells and organs can be helpful to compensate for the destruction of their lungs. More studies are mandatory to address the beneficial effects of stem cell therapies in patients with chronic pulmonary diseases affected by viral infections.

## Data Availability

Not applicable.

## Abbreviations

^+^SSRNA: Positive-single strand ribonucleic acid
ACE-2: Angiotensin-converting enzyme-2
ADAM17: A Disintegrin and Metalloprotease 17
ARDS: Acute respiratory distress syndrome
BM-MSCs: Bone marrow-derived mesenchymal stem cells
CCL2: C–C Motif Ligand 2
CD147: Cluster of differentiation-147
CD4+: Cluster of differentiation-4+
CO2: Carbon dioxide
COPD: Chronic obstructive pulmonary disease
COVID-19: Coronavirus-19
CXCL10: C-X-C Motif Chemokine Ligand 10
E: Envelope
ECM: Extracellular matrix
EGFR: Epidermal growth factor receptor
ERGIC: Endoplasmic Reticulum Golgi Intermediate Compartment
ESCs: Embryonic stem cells
HRV: Human rhinovirus
IFNs: Interferons
IgA: Immunoglobulin A
ILs: Interleukins
iPSCs: Induced pluripotent stem cells
IRF-7: Interferon Regulatory Factor-7
M: Membrane
MCP-1: Monocyte Chemoattractant Protein-1
MERS: Middle East Respiratory Syndrome
MHC: Major Histocompatibility Complex
MSCs: Mesenchymal stem cells
NF-ƙB: Nuclear Factor Kappa Light Chain Enhancer of Activated B cells
NSPs: Non-structural phosphatase
O2: Oxygen
ORFs: Open reading frames
PRRs: Pattern recognition receptors
ROS: Reactive oxygen species
S: Spike
SARS: Severe acute respiratory syndrome
SARS-CoV-2: Severe Acute Respiratory Syndrome Coronavirus 2
TGF: Tumor growth factor
Th17: T helper 17
TLRs: Toll-like receptors
TMPRSS2: Transmembrane Protease Serine 2
TNF: Tumor necrosis factor
TSLP: Thymic stromal lymphopoietin
